# Complete Genome Sequence of *Curtobacterium* sp. Isolated from Surface-Sterilized Germinating Alfalfa Seeds

**DOI:** 10.1128/mra.00905-21

**Published:** 2022-02-10

**Authors:** K. Karl Compton, Roderick V. Jensen, Kevin Lahmers, Birgit E. Scharf

**Affiliations:** a Department of Biological Sciences, Virginia Polytechnic Institute and State University, Blacksburg, Virginia, USA; b Department of Biomedical Sciences and Pathobiology, Virginia-Maryland College of Veterinary Medicine, Virginia Polytechnic Institute and State University, Blacksburg, Virginia, USA; Montana State University

## Abstract

We reported the complete genome sequence of a member of the pathogenic *Curtobacterium* genus. The sample includes a circular 3,955-kb chromosome, a 164-kb megaplasmid and a 42-kb plasmid. This strain was isolated from surface-sterilized alfalfa seeds.

## ANNOUNCEMENT

The genus *Curtobacterium* is known for its pathogenic members that cause soft rot of various plant species, e.g., poinsettia, sugar beet, grains, and dry beans (1–3). However, many other members are important decomposers and plant mutualists (3–5). Despite the many pathogenic isolates of *Curtobacterium*, a considerable number are plant growth-promoting bacteria (PGPB) or show potential for the bioremediation of metals (5, 6). *Curtobacterium* sp. strain TC1 is the latest addition to this genus of understudied but ecologically important organisms.

Strain TC1 was isolated from alfalfa (*Medicago sativa* L. var. Guardsman II registration number CV-203, PI 639220) seeds harvested in 2012 and stored at 4°C. Seed batches were washed three times with sterile water, surface sterilized with 3% hydrogen peroxide, and rinsed three times with sterile water. The seeds were then incubated in water for 24 h at 30°C in the dark. Streaking 10 μL of the liquid onto plates containing 1.5% agar, 0.5% tryptone, 0.3% yeast extract, and 0.087% CaCl_2_ × 2H_2_O (TYC) yielded single *Curtobacterium* sp. TC1 colonies.

Genomic DNA for both sequencing technologies was isolated from a *Curtobacterium* sp. TC1 culture grown at 30°C in TYC broth using a phenol-chloroform extraction protocol (7). Illumina MiSeq sequencing with Swift Biosciences library prep kit was first used and generated 4,030,212 paired-end reads. Reads were quality filtered and trimmed using BBduk in Geneious Prime 2021.2.2. Initial assembly of paired reads was performed *de novo* with the Geneious assembler on medium-low sensitivity/fast. The *N*_50_ was 43,861, and the average coverage was about 120×.

Oxford Nanopore sequencing utilized a 9.4.1 minION flow cell and SQK-LSK109 ligation sequencing kit. A total of 537,904,841 bp was generated across 20,000 reads. Guppy 4.4.1 was used for base calling, and adaptors were trimmed with Porechop 0.2.4. The mean read length was 26,845 and the read length *N*_50_ was 75,214. Nanopore data were assembled using Flye in Geneious and high-quality Illumina reads were used in Geneious to correct Nanopore sequencing errors. This combined assembly generated a circular chromosome of 3,954,930 bp with 70.7% GC content and two circular plasmids; 163,762 bp with 65.6% GC content named pTCL, and 41,985 bp with 67.8% GC content named pTCS. Annotation was performed by the NCBI Prokaryotic Genome Annotation Pipeline 5.2 (8–10). The annotation revealed 3,910 protein-coding sequences, 63 RNA genes (48 tRNAs), four of each ribosomal RNA, and 42 pseudogenes. The annotation of the plasmids revealed 50 protein-coding sequences in pTCS and 166 coding sequences in pTCL. Numerous flagellar structural, assembly and motor proteins are present, suggesting the possibility of motility in this organism, yet no obvious chemotaxis genes were identified. pTCL appears to contain a gene encoding a WXG 100 family protein, which is predicted to be secreted by type VII secretion systems (11). While no type VII secretion system genes were identified, genes encoding type II and type IV secretion systems were annotated in the chromosome. This is evidence that the bacterium may be capable of pathogenesis.

### Data availability.

The chromosome is accessible under accession number CP081964, pTCL under CP081962, and pTCS under CP081963. Raw reads from Illumina sequencing are accessible under SRX11895606, and raw reads from Nanopore sequencing are accessible under SRX11895607.
